# Supplementary material for: Are carbon emissions trading and green financial instruments synergistic? -Comprehensive quantitative research based on content analysis
Source: PLoS One. 2024 Mar 7;19(3):e0298601. doi: 10.1371/journal.pone.0298601 (PMC10919601; doi:10.1371/journal.pone.0298601)
Supplement: S1 Data — (ZIP) [file pone.0298601.s001.zip › Data/地级市Z_F工作报告.zip!/╒π╜¡/╓█╔╜╩╨/└┤╫╘╣1⁄2╓┌║┼í╛┬φ┐╦╩2╛▌═°í┐_2017.docx]

各位代表：

现在，我代表市人民政府向大会报告工作，请予审议，并请政协委员和其他列席会议的同志提出意见。

一、过去五年及2016年工作回顾

过去五年，我们全面贯彻党的十八大和十八届三中、四中、五中、六中全会精神，以习近平总书记系列重要讲话精神特别是视察舟山重要指示为指引，在省委、省政府和市委的正确领导下，在市人大、市政协的监督支持下，紧紧团结和依靠全市人民，凝心聚力，克难攻坚，推进国家战略落地实施，实现经济社会平稳较快发展，为新区开发建设奠定了坚实基础。

新区发展框架全面拉开。新区发展规划获国务院批复，舟山江海联运服务中心、舟山港综合保税区等获批实施，海洋产业集聚区等五大经济功能平台初步发挥主力军作用，海投、交投等四大国资公司组建运作。新区建设三年行动计划顺利实施，波音737完工和交付中心等重大项目成功落地，绿色石化基地、宁波舟山港主通道、鼠浪湖矿石中转码头、大陆引水二期、浙能六横电厂等重大项目开工建设或建成投用，累计完成固定资产投资4758亿元，年均增长22.3%。新区发展规划明确的国家支持政策已有19项落地，新增12万亩建设用地规划指标，争取到中央和省补助资金400多亿元，引进市外资金1600多亿元。

全市综合实力大幅提升。地区生产总值年均增长9.9%，增速位居全省首位；人均超过1.6万美元，列全省第三。一般公共预算收入年均增长9.5%。海洋经济比重由68.6％提高到70.2%。船舶工业产值超过千亿元，7家企业入选国家造船“白名单”。舟山江海联运服务中心建设实质性启动，宁波舟山港一体化稳步推进，舟山港域货物吞吐量达到4.26亿吨，江海联运运量达到1.6亿吨。国家蓝色旅游示范基地获批，纳入国家全域旅游示范区创建名录，成功举办两届国际海岛旅游大会并成为大会永久会址。渔农业转型升级扎实推进，国家远洋渔业基地获批，远洋渔业产量达到54万吨，占水产品总产量的比重从14.8%提高到28.3%。海洋科学城初具规模，世界首个多端柔性直流输电工程建成启用，高新技术产业占规上工业增加值比重从12.4%提高到44.1%。金融发展水平稳步提升，成功发行全国首单非上市企业公司债、全省首单租赁资产证券化产品，融资总量近2700亿元，年均增长13.9%。“智汇群岛·创新引领”5313行动计划成效明显，全市人才资源突破23.5万人。

改革开放工作走在前列。大力推进重点领域和关键环节改革，承担国家级改革试点12项、省级56项。“大市场”监管、陆上综合执法、海洋综合执法等改革走在全国前列，“项目中心制”、排污许可证“一证式”管理等多项改革举措在全省推广。舟山港综合保税区发展水平跻身全国中游，全市口岸开放面积达到1344.8平方公里，低空空域开放规划成功获批。创新保税燃料油供应监管模式，持续推进外锚地供油，突破同一税号下调和、跨关区港区直供、“一船多供”等政策。

城乡统筹步伐不断加快。本岛“南生活、中生态、北生产”的城市格局基本形成，城市化率达到67.5%。海陆综合交通网络不断完善，公交出行分担率达到18%，形成主要岛际水上航线2小时交通圈。城乡一体化政策体系日趋完善，渔农村股改完成率达到99%，建成渔农村特色精品社区100个，在全省率先实现美丽乡村创建全域化，城乡统筹发展水平列全省第四。“三改一拆”“五水共治”“一打三整治”等工作深入开展，完成“三改”面积1215万平方米、拆违面积682万平方米，实现本岛城乡供水一体化，治理黑臭河70条118公里，全面落实伏季休渔制度，整治拆除“三无”船只2000多艘。节能减排任务全面完成，空气质量保持全国领先。成功创建国家环保模范城市、国家节水型城市，中街山列岛和马鞍列岛获批成为国家级海洋牧场示范区、国家海洋公园。

社会民生事业持续改善。社会民生支出累计达676.4亿元，比上五年翻一番。城乡居民收入分别达到4.8万元和2.8万元，年均增长9.6%和10.6%，城乡居民收入差距缩小到1.71：1。城镇新增就业6.4万人，转移渔农村劳动力1.6万人，城镇登记失业率控制在3%以内。在全省率先实现养老保险和医疗保险城乡统筹，基础养老金标准由每人每月70元提高到145元，城乡居民大病医疗保险实现制度和人群双覆盖，大病保险报销比例提高9.5个百分点。居家养老服务照料中心覆盖全市社区，社会救助标准实现城乡一体化。安居工程稳步推进，完成危旧房改造810幢，建成保障性住房35209套。大力推进教育公平，义务教育均衡化基本实现，等级幼儿园比例达九成以上，新区旅游与健康职业学院获批设立，舟山职业技术学校新校区建成投用，浙江海洋学院升格为浙江海洋大学，浙江大学海洋学院建成开学。成功创建省卫生强市，市级医疗资源布局优化调整，国家卫生县城实现全覆盖，城乡医疗卫生服务体系基本建立，人均期望寿命由78.35岁提高到79.58岁。在全国首创“淘文化”平台，建成市海洋文化艺术中心、普陀保利大剧院、市体育场等大型文体设施，渔农村文化礼堂达到151家，形成中国海洋文化节、观音文化节、东海音乐节等节庆品牌。广电、新闻出版、档案、史志、文物和非遗保护等事业取得新成绩。工青妇等群团组织作用进一步发挥，民族、宗教、外事、侨务、老龄、儿童、红十字、慈善和残疾人等事业全面发展，海防、人防等工作不断加强。群众安全感满意率稳居全省前列，连续12年被省委、省政府授予“平安市”称号，成功夺得“平安金鼎”。荣膺全国“双拥模范城”七连冠。

政府自身建设切实加强。注重提高政府工作人员法治思维，全面加强依法行政，建立府院联席会议制度，法治政府和服务型政府建设扎实推进。“三强三优”行政体制创新顺利实施，市政府工作部门、市级事业单位分别精简26.5%和24.8%。加快简政放权步伐，基本实现市县同权，网上行政服务实现四级联动，“12345”新区社会公共服务和监督平台上线运行。自觉接受人大、政协和社会各界监督，认真执行市人大及其常委会决议，主动向政协通报重大事项，781件人大建议、1595件政协提案全部办复。重视发挥新区决策咨询委和新区顾问作用，形成电视问政、新区聚焦、网络问政和市民巡访全媒体舆论监督机制。认真落实全面从严治党决策部署，扎实开展党的群众路线教育实践活动、“三严三实”专题教育和“两学一做”学习教育，从严从实整治“四风”突出问题，公共项目建设中心、造价中心、审计中心有效运行，完成行政单位公务用车制度改革，“三公”经费支出逐年压缩，一批违纪违法案件得到严肃查处。

2016年，我们按照市委“树标杆、补短板、求突破、走前列”大行动部署，围绕“跑出发展加速度、树立新区新形象”要求，创新突破，较好地完成了年度目标任务，地区生产总值、固定资产投资等主要经济指标增速位居全省前列，实现了“十三五”发展良好开局。

这一年，我们加速国家战略落地。全力申报中国（浙江）自由贸易试验区，总体方案获国务院批复。持续推进海上保税燃料油供应，直供量超过100万吨，居全国港区第二位。综合保税区实现贸易额450亿元，增长1倍以上，船配进出口值达1.73亿美元，衢山分区封关运行。制定实施舟山江海联运服务中心行动方案，谋划推进74个重大项目，黄泽山油品储运贸易基地开工建设，小洋山北侧合作开发达成共识，推动特定航线江海通航船舶建造规范和检验暂行规则制定实施，2万吨级江海直达散货船完成开工准备，江海联运公共信息平台上线运行，甬舟铁路列入国家中长期铁路网规划。超常规推进绿色石化基地建设，规划环评通过审查，基地总体发展规划获批实施，4000万吨/年炼化一体化项目申请报告和项目环评均已通过评审。

这一年，我们全力推进项目建设。创新完善招商管理体制，成立新区招商局和新区企业投资服务中心，实际利用市外资金510亿元，浙商回归引进项目省外到位资金203.5亿元，千岛中央商务区、中澳现代产业园等项目引进落地。实施“项目中心制”，开展“比学赶超”活动，157个市重点项目扎实推进，超额完成年度投资计划。加快推进观音文化园、国家远洋渔业基地、329国道改建等重大项目，500千伏联网输变电工程、大陆引水三期、国际海岛旅游大会永久会址等重大基础设施项目开工建设。

这一年，我们大力扶持产业发展。推进供给侧结构性改革，实施企业减负政策，累计为企业减负19.6亿元。扎实推进“五批十大”项目，工业和技改投资保持较快增长。出台指导性意见，促进建筑业和房地产业健康发展。推进“四换三名”工程，“浙江制造”品牌认证实现零突破。召开新区企业家大会，隆重表彰一批优秀企业和企业家，激励企业家创新创业。入围国家首批海洋经济创新发展示范城市，积极实施蓝色海湾整治工程。大力开展“三增”“三减”“三严”渔业安全专项行动，促进海洋渔业优化结构，392艘渔船进入拆解程序。智慧海洋建设取得进展，深化与中船工业、中船重工、中电科技等企业战略合作，海洋数据中心、卫星通信、北斗应用等项目启动建设。有序推进电子商务产业集聚，建成电商园区8个、渔农村特色电商村8个。制定出台科技、人才发展新政，实施“双倍增”行动、科技助飞计划，规上工业高新技术产业产值和增加值分别增长17.9%和16.8%，世界首台3.4兆瓦海洋潮流能发电机组发电并网。加快建设朱家尖禅意小镇、沈家门渔港小镇和定海远洋渔业小镇，培育发展一批市级特色小镇。全力防范“两链”风险，不良贷款率达到省控目标以下。

这一年，我们不断深化改革创新。以打造全省“三最”城市为目标，深化“四张清单一张网”建设，企业项目审批服务流程缩短到48个工作日。深入推进户籍制度改革，实施新型居住证制度。深化渔农村“三权”改革，全面开展土地承包经营权确权登记颁证。健全“三位一体”渔农民合作经济组织体系，完成三级渔农合联组建。推进国资和投融资体制改革，5家企业挂牌新三板。创新土地利用方式，消化批而未供土地4900亩，处置供而未用土地7949亩，实施岱西盐场垦造耕地工程，获评“全国国土资源节约集约模范市”称号。

这一年，我们整治提升城乡环境。全力推进“三改一拆”“五水共治”“一打三整治”工作，制定实施中心城区污水管网建设三年行动计划，新建、修复管网58公里。加快百里滨海大道建设，推进交通治堵工程，新增专用停车位3332个。全面启动小城镇环境综合整治行动。创建美丽海岛示范线7条、综合示范岛6个，圆满承办全省美丽乡村和农村精神文明建设现场会。启动国家园林城市及森林城市创建，新增绿道网29公里，打造优质综合公园3个、示范街3条、绿化美化示范路3条。开展百日环保执法专项行动，全面推进污染防治和环境监管，空气质量综合指数全国领先。

这一年，我们持续改善民生福祉。认真抓好民生实事，财政民生支出增长13.4%。推进海洋捕捞渔民养老保障工作，出台支出型贫困家庭救助暂行办法。成立新区综合养老服务中心，基本形成15-20分钟养老服务圈。抓好“菜篮子”工程，实施减少流通环节、加强直供店和社区菜店建设等15项举措。全面推动教育基本现代化县（区）创建，普惠性幼儿园覆盖面达到87.9%，职业教育办学条件进一步完善。推进“海洋健康城市”建设，深化医改和公立医院综合改革试点工作，设立网络医院远程医疗服务中心6个。建成城市书房5家，“淘文化”产业平台入驻企业600余家。圆满完成G20杭州峰会等重大活动维稳安保工作，加强渔业、交通、危化等重点领域安全生产工作，安全生产事故起数、死亡人数实现下降。

各位代表，过去五年的成绩来之不易。这是省委、省政府和市委正确领导，全市百万军民共同奋斗的结果。在此，我代表市人民政府，向在各行各业辛勤劳动的全市人民，致以崇高的敬意！向人大代表、政协委员，向驻舟人民解放军和武警部队官兵，向在舟部省属单位，向各民主党派、工商联、人民团体和社会各界人士，向所有投资者、建设者，向关心和支持我市现代化建设的港澳台同胞和海内外朋友们，表示衷心的感谢！

这五年，我们深切体会到：只有始终牢记国家赋予的神圣使命，自觉地把新区发展融入国家战略的大局中，找准定位，才能抓住新区发展的历史机遇；只有积极寻求新区发展的突破路径，始终聚焦我们既该干又能干成的大事，砥砺前行，才能干出新区发展的蓬勃气象；只有牢牢把握创新发展的时代脉搏，始终在改革开放中充分发挥先行先试优势，走在前列，才能强化新区发展的引领作用；只有牢固树立以人民为中心的发展理念，始终把增强群众获得感作为新区发展的落脚点，共建共享，才能绘就新区发展的壮美蓝图。

我们也清醒地看到，我市经济社会发展中还存在不少困难和问题，政府工作还存在许多不足：经济总量和增速离目标要求还有差距，与国家级新区地位还不相匹配；多重国家战略加快落地实施，但有效的突破路径和创新方法不多；推动新区跨越发展的重大项目还比较缺乏，科技、人才、交通等要素保障仍然薄弱；新主导产业支撑作用尚未显现，传统产业发展仍面临较大困难，新旧动能转换的任务还很艰巨；城市能级、品质和综合竞争力还不高，国际化的短板尤其明显；民生保障、社会治理等领域还存在薄弱环节，群众获得感还不强；各级政府及工作人员的思想理念、工作方式和能力素质，还不能完全适应新区快速发展的要求，一些政府工作人员不作为、慢作为、乱作为现象依然存在。我们一定高度重视这些问题，采取更加有力的举措，认真加以解决。

二、今后五年的工作目标和主要任务

今后五年政府工作的指导思想是：高举中国特色社会主义伟大旗帜，坚持以邓小平理论、“三个代表”重要思想和科学发展观为指导，深入贯彻习近平总书记系列重要讲话精神和对舟山工作的重要指示，坚持“五位一体”总体布局、“四个全面”战略布局和新发展理念，深入实施新区发展规划，认真落实市第七次党代会决策部署，牢记势在必行，敢于勇立潮头，全面推进新区开发建设，早日把舟山建设成为自由贸易港区、海上花园城市。

今后五年政府工作的总体目标是：根据市第七次党代会“聚焦两重点、实现三提升、争取四翻番”的要求，到2021年，实现地区生产总值2000亿元，年均增长10%以上；海洋生产总值1400亿元，年均增长12%以上；规上工业增加值800亿元，年均增长14%以上；累计完成固定资产投资1万亿元，年均增长15%以上；全市一般公共预算收入达到180亿元，年均增长10%；城镇和渔农村常住居民人均可支配收入分别达到7.5万元和4.5万元；研究与试验发展经费支出占地区生产总值比重达2.5%以上。全力打造我国自由贸易港区先行区，浙江乃至长三角地区海洋经济发展的增长极，群岛型、国际化、高品质的海上花园城市，在全省率先高水平全面建成小康社会。

根据目标要求，今后五年政府工作的主要任务是：

（一）全面改革开放，打造我国自由贸易港区先行区

坚持先行先试，以中国（浙江）自由贸易试验区、舟山江海联运服务中心等国家战略为牵引，深度融入“一带一路”和长江经济带，打造国际一流的综合枢纽港，全面构建起面向国际、联通江海、辐射长三角的对外开放体系，努力走在我国海洋开放的最前沿。

探索建设舟山自由贸易港区。推动油品等全产业链投资便利化和贸易自由化。以保税燃料油供应为突破口，加快拓展国际船舶管理服务，提升国际航运服务功能，保税燃料油供应量达到600万吨。完善油品储备体系和储运投资便利化举措，建设国际油品储运基地；扩大油品加工领域投资开放，落实产业配套政策，形成5000万立方米罐容总规模、4000万吨油品储存规模、2000万吨炼油能力。提升油品资源配置能力，大力发展油品内外贸分销网络和交易市场，推进油品现货交易，建设东北亚保税燃料油交易中心。探索油品期货交易业务，建设国际油品交易中心。依托鼠浪湖、马迹山矿石中转码头，打造国际配矿贸易中心，形成区域性的矿石信息中心和价格形成中心，建设国际矿石中转基地。实现国际化大宗商品现货交易额1000亿元以上。创新大宗商品贸易自由化、便利化管理体制机制。实施自贸试验区内海关特殊监管区域“一线放开、二线安全高效管住”，探索建立货物状态分类监管模式。扩大金融服务领域开放，争取推动人民币跨境使用、计价结算等政策创新，支持中资、外资银行设立分支机构，鼓励境内外投资者设立融资租赁企业、保险公司、证券公司等其他非银金融机构，促进跨境贸易、投融资结算便利化。加快形成贸易便利化的制度规范。打造法治化、国际化、便利化营商环境。构建权责明确、管理高效、运转协调的自贸试验区行政管理体制，组建精简高效的统一行政审批机构，实行国际贸易“单一窗口”服务模式。建立完善自贸试验区行政管理权力清单和责任清单，实施外商投资准入前国民待遇加负面清单管理制度。加快推进综合行政执法、知识产权综合管理、商事案件专业化审理等制度改革，完善法律服务体系。

加快建设舟山江海联运服务中心。强化与自由贸易港区融合发展，切实提升江海联运综合枢纽港功能。加快核心港区开发，进一步完善江海联运集疏运体系，打造铁矿石、油品、煤炭和集装箱等七大基地，增强大宗商品多式联运和储备加工能力。提升江海联运公共信息平台功能，打造国内权威的江海联运数据平台和航运电商平台。加快江海直达系列船型研发和专业化船队建设，形成规模化、集约化经营。建设新城现代航运服务集聚区，做大做强中国（浙江）大宗商品交易平台，不断增强国际海事服务基地对外竞争力。加强与沿江、沿海、省内内河和21世纪海上丝绸之路沿线港口合作，尽快发挥江海联运枢纽和对外开放桥头堡作用。全市江海联运重大项目完成投资2300亿元，万吨级以上泊位超过90个，港口货物吞吐量达到6亿吨，江海联运运量达到2.8亿吨，江海联运指数影响力明显提升。

推进高水平、高质量改革开放。围绕“最多跑一次”目标，全面推进“放管服”改革，深化“四张清单一张网”建设，实行企业投资项目承诺制，落实服务型政府建设“1113”行动计划，打造全省“三最”城市。深化投融资体制机制改革，全面推进市属国有企业整合提升，支持企业股份制改造，接轨多层次资本市场，实现企业上市新突破。实施最严格、最高效的资源要素管理制度，深化以亩产效益为导向的资源要素差别化配置改革，健全阶梯价格制度。依托中澳现代产业园建设，积极落实中澳自贸协定，打造舟山中澳地方经济合作示范区。深化与澳门及葡语系国家合作，加快推进国家远洋渔业分基地建设。依托浙台（舟山普陀）经贸合作区，打造进境水果集散基地，拓展对台贸易。主动对接长三角城市群发展规划，加强与上海合作，深化大小洋山区域综合开发。积极参与义甬舟开放大通道建设，主动融入宁波都市圈，加强基础设施互联互通和资源整合，打造六横义甬舟合作共建区，推动区域合作和同城化建设纵深发展。

（二）加快创新驱动，打造浙江乃至长三角地区海洋经济发展的增长极

围绕海洋强国和创新驱动战略，以深化供给侧结构性改革为主线，打好“拆治归”转型升级系列组合拳，推动海洋产业集群化、集聚化、特色化发展，加快形成绿色石化、船舶与海工装备、港贸物流、海洋旅游四大千亿级产业和现代航空等若干百亿级产业。到2021年，海洋经济生产总值占全省和长三角地区的比重分别达到15%和5%以上，海洋经济示范引领作用更加明显。

培育发展千亿百亿产业集群。绿色石化产业。建设绿色石化基地，推动石化中下游产业集约发展，延伸发展高新材料和精细化工，力争产值突破1600亿元。船舶与海工装备产业。建设船舶与海洋工程装备国家新型工业化产业示范基地，重点突破绿色节能船舶、高技术高附加值船舶和海洋工程装备的研发制造，培育发展船舶研发设计、融资租赁、信息化等产业，实现总产值1500亿元。港贸物流产业。建设国际物流枢纽岛，大力发展大宗商品中转、储运、配送和交易，进一步提升集装箱中转运输能力，培育发展现代航运服务业，力争总产出超过1100亿元。海洋旅游产业。创建国家全域旅游示范区，不断放大国际海岛旅游大会品牌效应，加快建设旅游示范岛、旅游风情小镇、国际旅游度假区、高等级旅游景区和旅游产业融合基地，大力培育旅游新产品新业态新模式，全市旅游接待量突破8800万人次，总收入超过1250亿元。现代航空产业。波音737完工和交付中心建成投产，干线飞机整机交付价值超过600亿元。发展航空装备及零配件制造等延伸产业，推进通用航空综合产业，培育设计研发、航空衍生、航空金融产业，航空产业园初具规模。海洋电子信息产业。建设国家级智慧海洋应用示范基地和海洋电子信息产业基地，发展海洋大数据产业园和海洋电子工业园，培育海洋大数据、海洋卫星通信、船舶电子等产业，实现总产出超过200亿元。特色渔农产业。建设国家远洋渔业基地和舟山渔场国家绿色渔业示范基地，做强做大水产品精深加工业和海洋生物医药产业，做实渔业全产业链，发展国家级海洋牧场，促进传统渔业捕捞、加工、贸易转型升级，加强渔场修复和综合管理，振兴舟山现代渔业，水产品加工业规上产值达到260亿元，远洋渔业基地经济总产出320亿元。促进现代农业园区和粮食生产功能区提档升级，构建美丽农业体系，大力发展民生农业、景观农业、特色农业，重点培育晚稻杨梅、生态茶叶等年产值上亿元特色产业。

拓展海洋产业集聚平台。强化政策引导，促进资源要素优化配置和集约利用，充分发挥经济功能区主平台作用。拓展海洋产业集聚区发展新领域，提升定海工业园区、普陀经济开发区、岱山经济开发区、金塘塑机螺杆特色产业集聚区等园区综合发展水平，推动汽配、螺杆等传统产业提质增量。加快发展舟山国际粮油产业园区、国际水产城、普陀海洋文化创意园等一批现代服务业集聚示范区。推进块状特色产业与新经济模式融合发展，加快建设一批省市级特色小镇。建设国家潮流能海上试验场，推动海洋新能源科技装备产业化，加快潮流能、波浪能示范工程应用。推进普陀区大健康产业园等建设，培育形成以高端养生、康体旅游为特色的综合型健康产业体系。建设军民融合特色产业园，打通“军转民、民参军”通道，促进军地在产业、科技、基础设施等方面深度融合，争创国家军民融合创新示范区。

构筑海洋经济创新高地。建设国家高新技术产业开发区，着力打造以海洋新兴产业为主导、高端要素集聚、创新创业环境一流的海洋科技创新中心。建设海洋经济创新发展示范城市，实现产业链协同创新和产业孵化集聚创新。做强海洋科学城，提升“一城、二园、三岛、四校、多载体”创新平台体系，谋划建设一批科创型特色小镇、离岸创新创业基地、开放式创业街区和高端众创空间，实现科技型企业“双倍增”。深入实施十大科技成果转化工程，完善科技大市场建设，加快科技创新成果转化。探索建设省海洋金融创新示范区，构建多元化金融服务体系，促进海洋产业与金融产业互动发展。落实《中国制造2025》和“互联网+”行动计划，加快培育新主体、新技术、新产品、新业态、新模式。全面实施标准化战略，加快质量强市、标准强市、品牌强市建设。打造海洋创新创业人才高地，落实新区人才新政，依托高校和科研院所，加快引进和培养各类高端人才和创新团队，加强新型智库建设，积极实施“千岛工匠”培育工程。

（三）坚持千岛共荣，打造群岛型、国际化、高品质的海上花园城市

强化规划引领，实施五年攻坚，全方位提升城市品质形象和综合功能，基本建成生态和谐的绿色城市、有机开放的共享城市、独具文化魅力的包容城市和永续发展的智慧城市，决不把脏乱差、污泥浊水、违章建筑带入全面小康，全力建设群众乐居乐业乐活的幸福家园。全市城市化率达到70%以上，社会民生发展主要指标位居全省前列。

完善群岛特色城市化格局。对标自由贸易港风范、海上花园城风韵和现代化城市风貌，坚持高起点规划、全域化布局，形成以本岛为核心、兼顾千岛开发的全域型海上花园城市建设体系。强化“带状组团”理念，高标准推进新城和小干岛、定海城区、普陀城区、普陀山-朱家尖、海洋产业集聚区等城市主体区域建设，有序开展旧城改造和古城保护，把中心城市打造成山、海、城、绿相互融合，组团式、开放式、紧凑型的“一城三带多组团”格局。坚持特色化、差异化发展，打造“休闲定海”“全景普陀”“多彩岱山”“曼妙嵊泗”，在全省率先实现美丽乡村示范县创建全域化。坚持“千岛特色城镇化”，推进六横、金塘、衢山小城市培育试点工作，加快中心镇、小城镇建设步伐，深化美丽渔农村、美丽海岛示范线、美丽示范岛建设，完善“一镇一特色”“一岛一主题”“一村一韵味”“一线一风景”的发展布局。充分发挥交通在城市建设中的基础性作用，建成宁波舟山港主通道，基本建成六横公路大桥，开工建设甬舟铁路，加快沪舟甬跨海大通道项目战略规划研究等前期工作，加速水上客运提质升级，完善岛际交通网络，全力构建多元、立体、无缝、高效的现代化综合交通体系。努力建成生产空间节约高效、生活空间宜居适度、生态空间山清水秀，“城在海上、海在城中、山海兼胜、人海和谐”的美丽家园。

提升城市国际化水平。坚持世界眼光、国际标准，制定具有舟山特色的城市国际化建设评价指标体系。对标国内外先进城市，推动城市建设和管理与国际接轨，打造国际空港，建设国际邮轮母港，积极构建海陆空一体的对外交通体系，不断提升金融商贸、公共服务、综合交通等城市功能的国际化水平。研究传承海洋历史文化名城的建筑美学，加强城市地标、城市建筑群、公共空间的精致化建设，实施城市标识、标牌等改造，打造具有海洋特色的城市品牌，全面展示国际化旅游城市形象。不断提高城市经济、人文环境和人才交流的国际开放度，建立多语种服务平台和服务团队，建设千岛中央商务区会展中心等一批标志性场馆，办好一批具有国际影响力的会展、赛事和经贸活动，积极拓展医疗卫生、教育办学、文化科研等领域全方位国际合作。建立健全涉外事务管理体系，探索建立海事法律服务集聚区，加快建设国际街区、国际社区、国际学校、国际医院，完善相关制度和配套服务，不断提升文化品质、市民素质、社会治理等软实力，构建具有国际吸引力的城市人居环境。逐步成为城市功能现代化、城市形象鲜明独特、对外开放高度自信、人文环境兼容并蓄，具有较强竞争力和影响力的国际化港口城市。

加强生态文明建设。坚持生态优先、绿色发展，切实保护好生态环境。强化生态红线和绿色引领意识，严格实施环境空间管制，完善环境监管体系，全面提升环境监控监测及预警能力。创建国家园林城市、森林城市，提升城市绿化覆盖率，推进海绵城市和地下综合管廊规划建设，构筑陆海统筹的生态景观体系。深入研究南部诸岛等岛屿的分类开发保护，合理利用岸线资源，科学推进定海湾、新城湾、普陀湾开发建设。严格实施《舟山市国家级海洋特别保护区管理条例》，推进海洋生态整治修复工程，创建全省海洋生态文明建设示范区。努力让新区的天更蓝、水更清、山更绿、环境更美好，成为美丽中国的海岛样板。

推进社会事业均衡发展。始终把保障和改善民生作为政府工作的出发点和落脚点。统筹资源配置，构建“10+20+30”公共服务设施体系。健全现代教育体系，全面实行学前教育招生“服务区”制度，做精做强中高职教育，大力发展民办教育，支持浙江大学海洋学院、浙江海洋大学等高校发展，教育发展主要指标达到全省中上水平。推进健康舟山建设，加快建设省级医学副中心，完成市二院、市中心血站迁建，深入推进医药卫生体制和公立医院改革，促进人口长期均衡发展，实现人群主要健康指标达到发达国家水平。优化市体育中心功能布局，打造体育综合体。实施更加积极的就业创业政策，健全养老、医疗、住房、最低生活等社会保障体系，实现社会保险由制度全覆盖向人员全覆盖转变，城乡居民基础养老金比“十二五”末提高50%以上，继续保持全省领先。坚持政府过“紧日子”，让人民过“好日子”，不断提高人民群众的获得感和幸福感，在共建共享改革发展成果上走在全省前列。

推动社会治理能力现代化。实施市民文明素质提升工程，加强道德教育，完善社会诚信体系，推进志愿服务常态化，全力创建全国文明城市。构建现代公共文化服务体系，促进公共文化产品和服务社会化发展，加强历史遗存和非物质文化遗产保护，创建全国海洋文化示范区。创新基层社会治理，推进“美丽群岛·幸福社区”建设，引导更多社会力量参与社区治理与服务。深化法治新区建设，开展“七五”普法，提高全民法治素养。全面构建海上花园城公共安全体系，牢牢守住生产安全底线，加快建立与国际接轨的多元化矛盾纠纷化解机制，提升突发事件应急联动处置能力，争创平安浙江示范区，打造全省乃至全国最安全城市。

三、2017年的工作安排

2017年是实施“十三五”规划的重要一年，是推进供给侧结构性改革的深化之年，也是新一届政府的开局之年。我们要紧紧围绕市委确定的工作主线，奋发作为，全力完成各项经济社会发展指标，切实做好各项工作。

建议全市经济和社会发展的主要预期目标为：地区生产总值增长10%以上；固定资产投资增长15%；一般公共预算收入增长8%；外贸出口额达到427亿元，占全国3‰以上；社会消费品零售总额增长10%；城镇和渔农村常住居民人均可支配收入分别增长8%和8.5%，节能减排降碳指标完成省定任务，以优异成绩迎接党的十九大和省第十四次党代会的胜利召开。

重点在六个方面实现突破：

（一）在探索建设舟山自由贸易港区上求突破。组建工作管理机构，科学编制实施方案。参与制订《中国（浙江）自由贸易试验区条例》（草案）等一批法规和规范性文件，加快梳理实施现有自贸试验区可复制、可借鉴政策。开展自贸试验区审批综合服务，率先建成国际贸易“单一窗口”，实施“先照后证、证照分离”及全程电子化登记改革，实行“一口受理”高效服务模式。加快建设国际海事服务基地，重点突破以油品为核心的大宗商品贸易交易。制定保税燃料油供应相关规则和实施办法，加快保税燃料油供应企业集聚，全年供应量达到200万吨。拓展外轮供应服务中心功能，供应货值突破3亿美元。建设鼠浪湖国际配矿贸易中心，配矿加工贸易量突破800万吨。加快建设黄泽山油品储运贸易基地，建成150万立方米油品储罐。积极发挥中国（浙江）大宗商品交易中心作用，组建保税燃料油、LNG、煤炭交易平台，引进一批中资、外资金融机构，探索油品、铁矿石等大宗商品现货挂牌交易，力争完成现货贸易额450亿元。

（二）在攻坚重大项目上求突破。把招商引资作为新区发展“一号工程”，统筹全市招商资源，打造国际化招商网络，组织实施“2553”计划，合力开展重大项目精准招商、专业招商、产业链招商，实现全市实际利用市外资金575亿元，浙商回归省外到位资金184亿元，实际利用外资3亿美元。深入实施“项目中心制”“三重”项目动态管理机制和“比学赶超”机制，加快推进168个省市重点项目。大力推进舟山江海联运服务中心建设，争取七大基地建设取得重大进展，完成江海联运直达首制船舶建造并投入运营，尽快筹建江海直达船队，搭建航运交易平台，探索开展船货交易，加强与沿海、沿江主要港口深度合作，江海联运运量达到1.8亿吨。继续推进绿色石化基地建设，炼化一体化项目一期全面开工，全年完成投资300亿元。开工建设波音737完工和交付中心项目，综合保税区空港分区封关运作。加速推进观音文化园、大陆引水三期等重大项目建设，加快甬舟铁路前期工作。强化项目基础要素保障，确保供地1万亩以上，消化批而未供土地4000亩，累计处置供而未用及闲置土地85%以上，加快实施岱西盐场等盐田复垦项目，推进大小鱼山、小洋山北侧、六横小郭巨等滩涂围垦造地工程。

（三）在加快产业培育发展上求突破。完善政策激励和要素保障，大力振兴实体经济，提振企业发展信心。加大“白名单”船企扶持力度，推进困难企业实施重组，创建船舶装备高新技术产业园区，积极打造外轮修造基地。编制石化、航空等产业集群发展规划，强化政策引导作用，重点扶持培育一批新的龙头骨干企业，延伸完善产业链和创新链，尽快形成产业规模。加快发展港贸物流业，着力突破航运咨询、海事法务、航运金融等高端航运服务业，强化重点航运企业帮扶，推进“小散弱”航运企业兼并重组，港口货物吞吐量达到4.5亿吨。加快打造大宗商品进口基地，做大做强铁矿石、船配等进口贸易，推动跨境电子商务发展，实现外贸进口额340亿元。加快创建国家全域旅游示范区，办好2017国际海岛旅游大会，高质量举办海岛旅游博览会，争创国家医疗旅游示范基地，重点打造一批旅游示范岛、旅游特色小镇和精品民宿，丰富海岛休闲度假等旅游产品，完善旅游咨询服务体系，力争旅游接待量和总收入分别突破5500万人次和800亿元。加快建设国家远洋渔业基地，完善远洋渔船卫生注册制，加强与澳门及葡语系国家的远洋渔业合作，力争中国鱿鱼交易中心挂牌。支持水产行业稳步发展，做强水产品精深加工业，提升产品附加值，促进传统渔业向集约化、高新化发展。健全新型渔农业经营体系，实施“互联网+渔农业”，培育一批电商村。提升改造市级蔬菜基地1000亩，打造定海、普陀美丽牧场，鼓励发展养殖业，积极发展休闲渔农业。开展渔船改造提升工程，压减捕捞渔船147艘。加强国家级海洋牧场建设，加大增殖放流，全面实施新的伏季休渔制度，打好“幼鱼保卫战”。加大科技创新力度，创建国家高新技术产业开发区。加快建设海洋经济创新发展示范城市，启动实施17个重点项目。争取舟山智慧海洋示范区纳入国家总体规划，引进智慧海洋行业龙头企业5家以上。加快建设国电海上风电、3.4兆瓦海洋潮流能发电、海洋耐腐蚀材料等项目。大力发展创客码头、普陀湾众创码头等众创空间，完善落实创业扶持政策，培育发展共享经济。加快13个省市级特色小镇建设。

（四）在建设海上花园城市上求突破。实施十大攻坚行动，总投资突破100亿元。加强城市交通治堵工作，加快推进百里滨海大道、宁波北仑峙头至舟山水陆联合通道等基础设施建设，打通“断头路”15条，拓宽瓶颈路13条，新建城市道路9条，建设28个公共停车场，新增2000个泊位。加大“三改一拆”力度，全面开展城乡危旧房治理、城中村改造和违法建筑拆除专项行动，改造完成C、D级危房139幢，完成10个城中村治理，拆除违法建筑100万平方米。持续深化“五水共治”工作，全面推行“河长制”，完成河库清淤（污）120万方，提标改造5座污水处理厂，新增污水管网50公里，修复改造175公里，中心城区污水处理率达到92%，在全省率先全面剿灭劣V类水体。加快综合管廊和排涝设施建设，打造海绵城市示范项目。强势推进小城镇环境综合整治，完成13个乡镇（街道）整治验收，组建城市管理工作机构。实施本岛主要城市道路中英文双语标识改造，开展主干道路户外广告专项整治，推进城市景观亮化工程，瑞金医院一期、绿城育华（国际）学校建成运行。全域推进美丽海岛建设，重点培育朱家尖漳州村等5个美丽渔村，完成省级美丽宜居示范村5个以上，提升美丽海岛示范线5条以上，打造万亩景观田园。推进垃圾分类处理工作，城区生活垃圾分类覆盖面达到80%，完成渔农村生活垃圾分类处理40个村以上。加快建设邻里中心等公共便民服务设施，打造定海芙蓉洲路等6条精品示范街。加快建设市植物园等9座城市公园，建成绿色游步道130公里。

（五）在深化改革创新上求突破。深化供给侧结构性改革，实施重点企业“一企一策”帮扶措施和跟踪服务，完善中小企业公共服务平台，深入开展小微企业“助保贷”业务，继续实施企业经营管理者素质提升计划；加快“智能制造”步伐，规范推行“弹性出让、先租后让、租让结合”工业用地机制，创新开展金融惠企业务，清理规范涉企收费措施，切实降低企业成本；建立健全过剩产能退出机制，倒逼“僵尸企业”市场出清；引导建筑业和房地产健康发展，住宅去化周期控制在省定目标以内；健全完善地方债务管理机制，积极防范化解“两链”风险，加大不良贷款处置力度，维护区域金融稳定。加快“最多跑一次”改革，推进“一窗受理、集成服务”，推行“互联网+政务”服务方式，突破部门数据共享瓶颈，完善基层服务中心建设，力争90%以上的特定事项或环节一次性办成，走在全省前列。深入推进综合行政执法体制改革，提升城市管理和公共服务水平。持续推进渔农村“三位一体”改革，健全渔农合联服务体系。完善居住证制度配套政策，依法保障流动人口合法权益。深化县域经济体制综合改革，开展规上工业企业统一评价。创新政府投融资机制，做大做强国资平台公司和产业基金，建立债务融资储备资源库，启动一批PPP项目。积极培育上市后备资源，大力推动优质企业股份制改造，争取2家企业进入辅导备案，3～5家企业在新三板挂牌。全面实施标准化战略，深化标准化综合改革。加强整合规范工作，完善陆海联合招拍挂出让模式。深化“信用舟山”建设，建立健全信用联合奖惩机制。

（六）在发展民生社会事业上求突破。实施更加积极的就业创业政策，强化创业孵化基地建设。统筹推进渔民养老保障工作，推进新区综合养老服务中心实体化运营。推动社会救助精准化，完善低保、特困人员供养、医疗救助、临时救助等制度。实施低收入渔农户收入倍增计划，开展十大帮扶行动，动态消除城乡零就业家庭。推进教育公平发展和质量提升，实施第三轮学前教育普惠化行动计划、民办民工子弟学校三年整治计划，开展义务教育资源跨区域、跨体制共享改革试点，标准化学校比例达到95%以上，积极稳妥完成高考综合改革试点工作，加快职业教育校企合作共同体建设。实施《健康舟山2030行动纲要》，加强健康教育，全面推进“双下沉、两提升”和分级诊疗，责任医生规范签约率达到30%，网络医院服务20万人次，县级以上医院预约挂号覆盖率达到100%。建成“一站式”公共文体服务平台，做强做大“淘文化”，办好浙江省第三届体育大会。加快推进数字档案室建设，大力发展民族、宗教、红十字、慈善、志愿服务等事业，支持工会、共青团、妇联、科协等群团改革与发展。创新军民深度融合发展机制，大力支持国防和部队建设。选优配强村社组织班子，做好村和社区组织、集体经济组织换届工作。持续深化平安舟山建设，构建乡镇（街道）综治工作、市场监管、综合执法、便民服务等四大平台，健全社会治安防控体系，推进社会公共安全视频监控联网和物联网建设，全力做好社会治安、金融安全、食品药品安全、防台防汛等工作，开展地质灾害综合治理清零专项行动，着重抓好危化品、船舶修造、消防安全、水陆交通、渔业生产等重点领域的安全监管，坚决维护社会和谐稳定。

按照民生实事“群众提、大家定、政府办”的理念，切实办好十方面为民实事。经过广泛征集，认真梳理了“菜篮子”工程、社会保障和帮扶、水环境综合治理、医疗惠民服务、美丽新区建设及人居环境改善、安居工程、群众出行、文体与教育发展、社会治安防控体系、网络服务惠民及行政审批等十方面56项候选项目，提请各位代表票决。我们将根据代表们的票决结果，全力以赴，确保完成。

各位代表！实现新的奋斗目标，必须加强新一届政府自身建设。我们将紧紧围绕建设服务政府、创新政府、高效政府、法治政府和廉洁政府的目标，全面提升服务水平，不断适应自由贸易港区建设法治化、国际化、便利化需求，推行全程“店小二式”服务，致力打造国际一流的营商环境；全面强化创新意识，不断提升干部政治素养，提高干部国际化专业化水平、开放性思维和改革创新能力，提振干部精气神，努力打造一支政治强、能力强、作风强的新区铁军；全面推进简政放权，不断创新政府管理服务方式，加大政府购买公共服务力度，全力打造高效、透明、便捷的政务服务环境；全面增强法治理念，不断提高依法行政水平，健全府院联席会议和行政复议、行政调解制度，深化政务公开，助力打造全省法治示范区；全面加强廉洁建设，不断提高政府系统拒腐防变和抵御风险能力，自觉接受市人大及其常委会的法律监督、工作监督、市政协的民主监督和监察委员会的监督，主动接受舆论监督和群众监督，促进权力规范、阳光运行，合力打造政治生态上的绿水青山。

各位代表！舟山正处在跨越发展的关键时期，我们深感责任重大。让我们更加紧密地团结在以习近平同志为核心的党中央周围，在省委、省政府和市委的坚强领导下，团结带领全市百万军民，牢记势在必行，敢于勇立潮头，为早日建成自由贸易港区、海上花园城市而努力奋斗！
